# Reliability of point-of-care circulating cathodic antigen assay for diagnosing *schistosomiasis mansoni* in urine samples from an endemic area of Brazil after one year of storage at -20 degrees Celsius

**DOI:** 10.1590/0037-8682-0389-2021

**Published:** 2022-02-25

**Authors:** Tereza Cristina Favre, Lilian Christina Nóbrega Holsback Beck, Fernando Schemelzer Moraes Bezerra, Carlos Graeff-Teixeira, Paulo Marcos Zech Coelho, Martin Johannes Enk, Naftale Katz, Ricardo Riccio Oliveira, Mitermayer Galvão dos Reis, Otávio Sarmento Pieri

**Affiliations:** 1 Fundação Oswaldo Cruz, Instituto Oswaldo Cruz, Laboratório de Educação em Ambiente e Saúde, Rio de Janeiro, RJ, Brasil.; 2 Universidade Federal do Ceará, Departamento de Análises Clínicas e Toxicológicas, Fortaleza, CE, Brasil.; 3 Universidade Federal do Espírito Santo, Centro de Ciências da Saúde, Unidade de Doenças Infecciosas, Vitória, ES, Brasil.; 4 Pontifícia Universidade Católica do Rio Grande do Sul, Laboratório de Parasitologia Biomédica, Porto Alegre, RS, Brasil.; 5 Fundação Oswaldo Cruz, Instituto René Rachou, Belo Horizonte, MG, Brasil.; 6 Instituto Evandro Chagas, Laboratório de Parasitoses Intestinais, Esquistossomose e Malacologia, Secção de Parasitologia, Ananindeua, PA, Brasil.; 7 Fundação Oswaldo Cruz, Instituto Gonçalo Moniz, Salvador, BA, Brasil.; 8 Universidade Federal da Bahia, Faculdade de Medicina, Salvador, BA, Brasil.; 9 Yale University, School of Public Health, Department of Epidemiology of Microbial Diseases, New Haven, CT, United States of America.

**Keywords:** Schistosoma mansoni, Point-of-Care (POC) testing, Circulating Cathodic Antigen (CCA), frozen urine, Kato-Katz, Helmintex®, Brazil

## Abstract

**Background:**

The World Health Organization recommends reliable point-of-care (POC) diagnostic testing to eliminate schistosomiasis. Lateral flow immunoassay that detects schistosome circulating cathodic antigen (CCA) in urine to establish prevalence thresholds for intervention in endemic areas is recommended. Stored urine may be useful if surveying at-risk populations is delayed or interrupted by unforeseen circumstances, such as the current COVID-19 pandemic. This study evaluated the manufacturer’s claim that *Schistosoma mansoni* infection can be reliably diagnosed in urine samples stored at -20°C for one year.

**Methods:**

Two-hundred-forty-two subjects from an endemic site in Brazil provided one urine sample each for testing with URINE CCA (SCHISTO) ECO TESTE® (POC-ECO) and one stool sample each for testing with Kato-Katz (KK) and Helmintex® (HTX) as a robust reference standard for infection status. At least 2 ml of urine from each participant was stored at -20°C; after one year, 76 samples were randomly selected for POC-ECO retesting.

**Results:**

The POC-ECO agreement between freshly collected and stored urine was inadequate considering trace results as positive (Cohen’s kappa coefficient κ = 0.08) and negative (κ = 0.36). POC-ECO accuracy was not significantly greater than that of routine KK (54%; 95% confidence interval: 42.1%-65.5%).

**Conclusions:**

The precision and accuracy of POC-ECO have to be optimized in both freshly collected and stored urine before it can be recommended for use in control programs in Brazil.

## INTRODUCTION

In 2020, the World Health Organization^1^ launched a new 2021-2030 “road map” to hasten the elimination of neglected tropical diseases (NTDs), including schistosomiasis, as part of the Sustainable Development Goals (SDGs) Agenda. One key measure to eliminate schistosomiasis as a public health problem by 2030 is to develop standardized point-of-care diagnostics^1^. The WHO currently recommends point-of-care immunochromatographic testing to detect schistosome circulating cathodic antigen in urine as an alternative to the routinely used Kato-Katz (KK) fecal thick smear^2^ - two slides from a single stool sample - to establish prevalence thresholds for varying treatment schemes and additional interventions in endemic areas (https://www.who.int/activities/enhancing-implementation-of-schistosomiasis-control-and-elimination-programmes Accessed 19 June, 2021). However, several studies have indicated a lack of reproducibility and the need for extensive and rigorous evaluation in different settings^3^ ^-^ ^5^.

One issue in POC-ECO performance evaluation is the manufacturer’s claim that urine samples can be stored at -20° C for one year before testing at room temperature (20-25° C). As pointed out by van der Hel *et al*.^6^, several population-based studies have collected and stored frozen urine samples, which can subsequently be used in reliable, validated assays. This is especially important given the impact of the COVID-19 pandemic on public health actions against NTDs^7^, which include delays in diagnostic testing and disruption of periodic treatment in schistosomiasis high transmission areas^8^. In Brazil, all 472 priority municipalities that had agreed to intensify schistosomiasis control under the Ministry of Health (MoH) 2019-2022 Action Plan have postponed their actions indefinitely, including treatment of infection carriers identified by single-sample KK in localities at the greatest risk^5^.

Stored urine was tested with different batches of POC-ECO distributed in Brazil by Eco Diagnóstica Ltda (http://ecodiagnostica.com.br/diagnostico-rapido/urine-cca-schisto-eco-teste/#detail-product Accessed 19 June, 2021), on the assumption that outcomes would not be significantly different from those obtained with freshly collected urine. Accordingly, Viana *et al*.^3^ used urine from 64 egg-positive patients that had been frozen at -80°C for an unspecified period. However, they did not report the outcomes of the freshly collected samples. Similarly, Grenfell *et al*.^9^ used urine from 27 egg-positive and 21 egg-negative individuals previously stored for two years at -20°C, but no outcomes from freshly collected urine were provided. In contrast, Graeff-Teixeira *et al*.^5^ showed unacceptably low specificity in POC-ECO results from freshly collected urine and one-year stored (-20°C) urine from a non-endemic area.

The present study forms part of a multicenter research project to evaluate the performance of POC-ECO in Brazil’s endemic settings and to produce recommendations for its inclusion in the MoH schistosomiasis elimination action plan. The study was conducted in nine representative localities following the same core protocol. Due to operational issues, the performance of POC-ECO for diagnosing schistosomiasis mansoni in the same urine samples (i) fresh after collection and (ii) frozen at -20°C for one year was evaluated in only one endemic locality, the results of which are presented here.

In Brazil, POC-ECO is commercially available as an Eco-Teste® (ECO Diagnóstica Ltda. Corinto, MG, Brazil), registered (MoH 80954880012), and approved by the Brazilian Health Regulatory Agency (Anvisa) for use as an auxiliary diagnostic tool for patients with clinical signs and symptoms of active infection. However, further performance validation is required for it to be used in surveillance and control actions in endemic areas. POC-ECO will be recommended to the MoH for use with stored urine only if outcomes are both accurate and precise, as described by Viera and Garrett^10^.

## METHODS

### Study Area and Sampling

This study was conducted in Jaguaritira (17°54’00.0” S; 42°13’58.8” W), a rural district in the municipality of Malacacheta with a population of approximately 900, located in the endemic area of Minas Gerais, Brazil^11^. The prevalence of schistosomiasis estimated by the KK (single sample, two slides) among schoolchildren (6-15 years-old) was 13% in 2015^12^. In May-June 2019, all children aged 11-13 years attending the middle years of upper secondary school in Jaguaritira, together with their families, were invited to participate in the multicenter study, the findings of which will be published in good time. All family members over two years old were eligible to participate after signing and/or oral assenting to the Free and Informed Consent Form, which included information that part of the collected material would be stored and could be retested for quality control. Exclusion criteria were self-reported pregnancy, clinical signs or symptoms of acute illness, and severe chronic diseases^5^. Each consenting adult and assenting child was asked to provide approximately 50 g of stool and 10 ml of midstream morning urine, which was stored in a refrigerator at 4°C until the following day. For egg detection, stool samples were assayed using both KK and HTX, the former as a routine test, and the latter as a highly sensitive, robust reference test^13^ ^,^ ^14^. The HTX procedure involved the dispersal of 30 g of stool in a Tween-ethanol solution, repeated filtration and sedimentation followed by incubation with paramagnetic particles, separation of *S. mansoni* eggs with a magnet, and staining of the resulting sediment with ninhydrin. The sediment was then spread over slides of filter papers for examination by optical microscopy. Although laborious and time-consuming, HTX significantly increases egg detection in stool samples from low-intensity transmission areas compared with routine KK^15^ ^-^ ^18^.

Freshly collected urine samples were assayed by POC-ECO (batch number: 180907091; expiry date: 30 August, 2020) following the Eco Diagnóstica (ED) instructions for transportation, storage, and use (Edition 001/2018, approved on 12 June, 2018). Two drops of urine were transferred to the well of the testing cassette and a reading was taken in the 21^st^ minute by two experienced technicians blinded to the results of the stool tests. The outcome was scored by grading the POC-ECO test line on a scale of 1 to 10 (G1-G10) and recoding into a visual score as follows: G1, negative; G2-G3, trace; G4-G10, positive^19^. The G-score outcome was unanimously determined by readers. At least 2 ml of each urine sample was stored in a freezer at -20°C to ensure reliability after at least one year. The freezer power supply was backed by a generator to prevent accidental defrosting of the samples, and the temperature was recorded daily with a maximum-minimum thermometer. The 242 fully compliant participants averaged 27 years of age and 7% had more than 100 eggs per gram of stool (epg) as estimated with KK (one sample, two slides). The prevalence of infection was estimated using KK, Helmintex (HTX), and POC-ECO, both with trace outcomes divided into positive (t+) and negative(t-); the values obtained were 20% with KK, 56% with HTX, 77% for POC-ECO t+, and 57% for POC-ECO t-; the prevalence of KK in combination with HTX (KK+HTX) was 58%.

The minimum sample size for evaluating POC-ECO test-retest reliability at one year was calculated as recommended by Bujang and Baharum^20^. Landis and Koch^21^ and Viera and Garrett^10^ have shown that in order to determine a sample size that differentiates between a Cohen’s kappa coefficient of *κ*=0.0 (no agreement) and moderate agreement (*κ*=0.5), at least 38 urine samples were required for a statistical power of 0.9 (90%) and a significance level of 0.05; however, the minimum sample size was multiplied by two (38 × 2 = 76), as recommended by Bujang & Baharum^20^. This sample size provided a statistical power of 0.8 (80%) and a *p*-value of less than 0.05, to differentiate between 50% (null hypothesis) and 80% (minimum acceptable value) sensitivity and specificity for a true prevalence of 60%^22^.

### Sample Processing and Testing

From 242 urine samples from fully compliant participants stored at -20°C in May-June 2019, seventy-six were randomly selected for retesting in May-June 2020 with POC-ECO (Batch 201907001; expiry date: 30/04/2021). The POC-ECO batch used with freshly collected urine was not used with stored urine because its expiry date (30 August, 2020) was close to the retest period. Both batches were used at approximately half of their shelf lives to avoid stability bias. For retesting, samples were defrosted and homogenized at room temperature (25°C), as recommended by the manufacturer, and assayed by two procedures in parallel. One was identical to that used with freshly collected urine and the other, named POC-ECO/FLT, was a 30-minute procedure that yielded a filtered sample with tenfold concentration for testing^9^ ^,^ ^23^. In this field-friendly method designed to optimize the CCA assay^24^, each urine sample (0.5 mL) was inserted into a Microcon-30kDa centrifugal filter unit with Ultracel-30 membrane (MRCF0R030, Merck Millipore Ltd., Ireland), centrifuged (GMCLab PMC880 mini centrifuge, Gilson, USA) at 2000 g for 30 min, and re-suspended in 0.05 ml of distilled water to obtain a ten-fold increase in the concentration of the original sample. Two drops (100 µL in total) of the filtered, concentrated sample were then transferred to the well of the POC-ECO testing cassette and read, as described earlier.

### Data Analysis

Data were entered into an Excel spreadsheet and transferred to Systat 13 (Systat Software Inc., USA) for statistical analysis. The POC-ECO results (negative/trace/positive) were expressed in absolute numbers and percentages with 95% confidence intervals (CIs), where non-overlapping 95% CIs indicated significant differences (*p* < 0.05) between percentages. KK was used in combination with HTX (KK+HTX) as the benchmark for infection status (egg-positive/egg-negative) because KK alone is unreliable for this purpose in areas where egg load is low in most infection carriers^15^. The following performance parameters were estimated: sensitivity or true-positive rate (probability that the test result was positive among egg-positives), specificity or true-negative rate (probability that the test result was negative among egg-negatives), and accuracy (overall probability that egg-negatives and egg-positives were correctly classified). These three parameters were calculated for both POC-ECO and POC-ECO/FLT, considering traces (G2-G3) as positive (t+) and negative (t-), and for KK using the MedCalc diagnostic test calculator (https://www.medcalc.org/calc/diagnostic_test.php Accessed 19 June, 2021). Agreement between the two POC-ECO tests was estimated using Cohen's kappa (*κ*) statistic: a *κ*-value below 0.60 indicated inadequate agreement^25^.

### Ethical considerations

The multicenter study protocol was approved by the Ethics Committee of the Oswaldo Cruz Institute (CEP-IOC), Fiocruz (CAAE: 82469417.8.0000.5248). 

## RESULTS

Of the 76 participants who provided stool and urine samples in May-June 2019 and whose urine samples were retested after one year of storage at -20°C, 45 (59%; 95% CI: 45.2%-71.4%) tested positive with KK and HTX combined (KK+HTX). The prevalence of infection estimated using KK and HTX alone was 13.2% (95% CI: 5.5%-24.0%) and 56.6% (95% CI: 42.7%-69.0%), respectively. The prevalence of infection from freshly collected urine was 78.9% (95%CI: 65.9%-88.1%) with POC-ECO t+ and 15.8% (95% CI: 7.4%-27.1%) with POC-ECO t-. After one year of storage at -20°C, the prevalence of infection was 18.4% (95% CI: 9.3%-30.2%) with POC-ECO t+, 4.0% (95% CI: 0.4%-12.0%) with POC-ECO t-, 85.6% (95% CI: 73.4%-93.0%) with POC-ECO/FLT t+, and 35.5% (95% CI: 23.2%-48.6%) with POC-ECO/FLT t-. Of the ten participants who tested positive for KK, two had 108 epg (moderate-intensity infection) and tested positive for POC-ECO from both freshly collected and stored urine. The remaining eight (10.5% 95%CI: 3.5%-21.4%) who tested positive with KK had 1-99 epg (low-intensity infection) and, of these, two tested negative with POC-ECO from freshly collected urine and four tested negative with POC-ECO from stored urine.

Table 1 shows the POC-ECO outcomes from freshly collected and stored urine among egg-positive and egg-negative participants, separately and together. Among the 31 egg-negatives, POC-ECO returned three negative results and 25 traces from freshly collected urine, and 28 negatives and three traces from stored urine. The filtration-concentration method (POC-ECO/FLT) detected three negatives and 17 traces in stored urine among the 31 egg-negatives. Among the 45 egg-positives, POC-ECO identified nine positives and 23 traces from freshly collected urine, and three positives and eight traces from stored urine. POC-ECO/FLT detected 16 positives and 21 traces among the 45 egg positives. The *κ*-values calculated from the results in Table 1 to estimate agreement between POC-ECO findings from freshly-collected and stored urine in all 76 samples were as follows: 0.08 (95% CI: -0.01- 0.16) if traces are considered positive (t+) and 0.36 (95% CI: 0.06- 0.67) if traces are considered negative (t-).

**TABLE 1:** Results of POC-ECO by infection status determined by a robust reference standard (KK+HTX) from freshly-collected urine and urine retested after being stored at -20°C for one year.

Infection status (KK+HTX)	POC-ECO outcome		Freshly collected urine*	Urine stored at -20°C for one year**	
			POC-ECO	POC-ECO	POC-ECO/FLT
Egg-negative	Positive	N (%)	3 (9.7)	0 (0.0)	11 (35.5)
		95% CI	0.8% - 28.6%	-	15.5% - 57.4%
	Trace	N (%)	25 (80.6)	3 (9.7)	17 (54.8)
		95% CI	56.6% - 93.5%	1.1% - 27.5%	31.1% - 74.9%
	Negative	N (%)	3 (9.7)	28 (90,3)	3 (9.7)
		95% CI	0.8% - 28.6%	70.0% - 98.1%	0.8% - 28.6%
	**Total**	**N (%)**	**31 (100)**	**31 (100)**	**31 (100%)**
Egg-positive	Positive	N (%)	9 (20.0)	3 (6.7)	16 (35.6)
		95% CI	7.4% - 37.1%	0.6% - 20.5%	18.7% - 53.7%
	Trace	N (%)	23 (51.1)	8 (17.8)	21 (46.7)
		95% CI	31.9% - 68.5%	6.0% - 34.6%	28.0% - 64.4%
	Negative	N (%)	13 (28.9)	34 (75.6)	8 (17.8)
		95% CI	13.6% - 46.8%	56.0% - 88.4%	6.0% - 34.6%
	**Total**	**N (%)**	**45 (100)**	**45 (100)**	**45 (100)**
All	Positive	N (%)	12 (15.8)	3 (3.9)	27 (35.5)
		95% CI	6.9% - 27.9%	0.3% - 12.5%	22.4% - 49.4%
	Trace	N (%)	48 (63.2)	11 (14.5)	38 (50.0)
		95% CI	48.2% - 75.5%	6.0% - 26.3%	35.4% - 63.6%
	Negative	N (%)	16 (21.1)	62 (81.6)	11 (14.5)
		95% CI	10.7% - 33.9%	67.8% - 90.5%	6.0% - 26.3%
	**Total**	**N (%)**	**76 (100)**	**76 (100)**	**76 (100)**

POC-ECO: point-of-care circulating cathodic antigen manufactured by Eco Diagnóstica Ltd; KK+HTX: combined Kato-Katz (one stool sample, two slides) and Helmintex® methods *tested with batch 180907091; **tested with batch 201907001; POC-ECO/FLT: filtration-concentration method. N: number of cases; CI: confidence interval.

**FIGURE 1:**
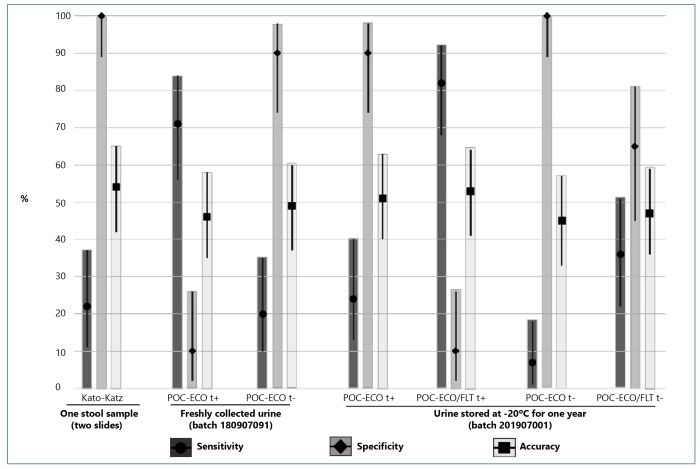
Performance of Kato-Katz, POC-ECO (batch 180907091) with freshly-collected urine, and both POC-ECO and POC-ECO/FLT (batch 201907001) with urine storePerformance of Kato-Katz, POC-ECO (batch 180907091) with freshly collected urine, and both POC-ECO and POC-ECO/FLT (batch 201907001) with urine stored at -20°C for one year, taking as reference the joint outcome of Kato-Katz and Helmintex® (KK+HTX). Percentage (%) values for sensitivity, specificity, accuracy and 95% Confidence Intervals (CI) are depicted on vertical lines. POC-ECO and POC-ECO/FLT results were calculated considering traces as positive (t+) and as negative (t-).

Figure 1 depicts the performance of POC-ECO (t+ and t-) with freshly collected and stored urine, POC-ECO/FLT (t+ and t-) with stored urine, and KK (one stool sample, two slides) using KK+HTX as a reference. Sensitivity ranged from a minimum of 6.7% (95%CI: 1.4%-18.3%) for POC-ECO (t-) in stored urine to a maximum of 82.2% (95%CI: 68.0%-92.0%) for POC-ECO/FLT (t+). The urine test sensitivity of POC-ECO (t+) with freshly collected urine and POC-ECO/FLT (t+) was significantly higher than that of KK (22.2%; 95%CI: 11.2%-37.1%), as can be seen from the non-overlapping 95%CIs. Specificity ranged from 9.7% (95%CI: 2.0%-25.8%) with both POC-ECO (t+) in freshly collected urine and POC-ECO/FLT (t+) to 100% (95%CI: 88.8%-100%) with POC-ECO/FLT (t-). Urine test specificity of POC-ECO (t+) with freshly-collected urine and POC-FLT (both t+ and t-) in stored urine was significantly lower than that of KK (100%; 95%CI: 88.8%-100%). Accuracy ranged from 46.1% (95%CI: 34.6%-57.9%) with POC-ECO (t+) in freshly collected urine to 52.6% (95%CI: 40.8%-64.2%) with POC-ECO/FLT (t+) in stored urine. No significant difference (*p* > 0.05) in accuracy was detected among the urine tests, as the 95% CIs overlapped in all situations, and there was also no significant difference in the accuracy of urine tests compared with KK (54.0%; 95%CI: 42.1%-65.5%).

## DISCUSSION

The agreement between POC-ECO results with freshly collected and stored urine (test-retest) was inadequate, with κ-values below 0.60^25^, regardless of whether traces were considered positive (t+) or negative (t). Among egg-negatives, POC-ECO outcomes in freshly collected urine were mostly trace (25 of 31; 80.6%), whereas in stored urine, POC-ECO outcomes were mainly negative (28 of 31; 90.3%) (Table 1). The poor agreement obtained (*κ* = 0.08) if trace is considered positive (t+) may be due to the use of batch 180907091 in freshly collected urine, which may yield unduly high levels of trace outcomes regardless of whether they were purchased from the ED or Rapid Medical Diagnostics (RMD), South Africa (Daniel Colley, personal information). However, the weak agreement (*κ* = 0.36) obtained if the trace is considered negative (t-) may be attributed to the disproportionately high levels of negative outcomes with POC-ECO batch 201907001 in stored urine from egg-positives.

Note also that the POC-ECO/FLT filtration-concentration procedure applied in our study to POC-ECO batch 201907001 in stored urine reduced the negative outcomes by 80.6 percentage points (pp), from 90.3% to 9.7% among egg-negatives, and increased the positive outcomes by 35.5 pp (from 0.0% to 35.5%). Surprisingly, trace outcomes increased by 28.9 pp (from 17.8% to 46.7%) with POC-ECO/FLT among egg-positive patients (Table 1). This contrasts with previous results^9^ with batch 170522062 (also from the ED) in stored urine, as traces obtained with POC-ECO/FLT in that study were all from egg-negative individuals identified by a robust egg detection method.

The discrepancies found between the POC-ECO/FLT performance in that study and ours may be due to the different amounts of urine that ED required to be dropped on the circular well of the test cassette: ≈150 µL (three drops from the test kit pipette) in the former study and ≈100 µL (two drops from the pipette) in this study. Grenfell *et al*.^9^ followed instructions from Edition 002/2017 of the ED leaflet included in the batch 170522062 package, which required “three drops or 100 µl”; this study followed instructions from edition 001/2018 of the ED leaflet included in both the batch 180907091 and batch 201907001 packages, which required “two drops or 100 µl.” ED performs only the final manipulation process, which consist of machine-cutting the reagent-impregnated sheets received from RMD into individual strips, manually inserting the strip into the test cassette and foil-pouching it under rigorous quality control protocol; all prior phases are performed by the technology holder, RDM. Since October 2017, the assay has required two drops of urine and no buffer^4^. However, the ED folder currently available on the Internet wrongly retains the requirement of three drops of urine for POC-ECO testing (http://ecodiagnostica.com.br/wp-content/uploads/2018/04/Folder-Schistosoma.pdf Accessed 19 June, 2021). These POC-ECO quality assurance and quality control issues reinforce previous concerns with Schisto POC-CCA^®^ from Rapid Medical Diagnostics (POC-CCA) both in freshly-collected^4^ ^,^ ^26^ and stored urine^3^ ^,^ ^5^. Moreover, results from earlier studies based on a discontinued version of POC-CCA requiring one drop of urine and one drop of buffer^16^ ^-^ ^18^ ^,^ ^27^ ^-^ ^32^ may not be comparable with those based on the new version of POC-CCA requiring two drops of urine and no buffer^3^ ^,^ ^4^ ^,^ ^24^ ^,^ ^33^ ^-^ ^35^.

POC-ECO accuracy, that is, the overall probability that egg-negative and egg-positive individuals are correctly classified, was no better than routine KK (one stool sample, two slides), as evaluated by a robust reference standard (KK+HTX) based on egg detection. This disappointing result occurred with both freshly collected and stored urine, regardless of whether traces were considered positive or negative (Figure 1). Note that the filtration-concentration procedure (POC-ECO/FLT) did not improve the accuracy of stored urine with either t+ or t-. However, this is not unexpected because the improvement in POC-ECO/FLT is designed to yield benefits in low-infection settings and post-treatment situations^9^ ^,^ ^23^.

The main limitation of the present study was that it did not include a direct comparison between the two POC-ECO batches at test and retest, to account for both batch-to-batch variation and the effect of urine storage. The current Eco Diagnóstica pamphlet on POC-ECO (http://ecodiagnostica.com.br/wp-content/uploads/2016/09/Bula-Urine-CCA-Schisto-1.pdf) states that “urine samples can be stored at -20°C for one year”, but provides no evidence or reference to support this claim. As the present study showed a significant difference between POC-ECO performance at test and retest, confirming previous findings from a non-endemic area^5^, the manufacturer should withdraw this currently unsupported claim from the pamphlet. Additional work is required to assess the effect of urine storage and to optimize POC-ECO precision and accuracy before making claims for its use with long-term stored urine.

A recently published study^36^ using the same batch of POC-CCA both in fresh and frozen urine (-20°C) after one year found a significant decrease in the positive rate, from 100% (95%CI: 98.0%-100.0%) to 74.4% (95%CI: 66.9%-80.7%), considering trace as positive (t+). This was ascribed to the degradation of CCA, which might have occurred because the study area was susceptible to recurrent power failure, leading to undue thawing of the urine samples. In the present study, however, the possibility of accidental freeze-thaw cycles of the samples could be ruled out due to the use of a power generator, ensuring daily minimum and maximum temperatures of approximately -20°C.

To our knowledge, no previous study in moderately endemic areas has compared the accuracy of recent POC-ECO batches (two urine drops and no buffer) with that of routine KK (one stool sample and two slides) using a robust egg-detection method as a reference. It should be emphasized that diagnostic accuracy is a prevalence-dependent parameter^37^; therefore, the values obtained in areas with different endemicities may not be comparable. Moreover, it is recommended that “true” prevalence be estimated using robust reference methods to provide a better reflection of the true disease status in the target population. As part of a multicenter study, the accuracy of the POC-ECO assay for diagnosing schistosomiasis mansoni is being evaluated in areas of different endemicities using the same POC-ECO batch and the same robust reference method. As pointed out by Graeff-Teixeira *et al*.^5^, rigorous diagnostic tests based on egg detection from larger amounts of fecal material or increased sampling are meant for reference purposes, and not for use in routine control programs. Thus, a highly accurate, urine-based schistosomiasis rapid diagnostic method would be more cost-effective than labor - and time-intensive stool microscopy, particularly in endemic settings with limited resources.

## CONCLUSION

This study showed that the POC-ECO assay is not reliable for diagnosing schistosomiasis mansoni in urine stored at -20°C for one year, given the inconsistencies found in its performance. Before much work can be done to clarify the precision and accuracy of POC-ECO in both freshly collected and stored urine, manufacturers must optimize the quality assurance and quality control measures.
